# COVID-19–Related Life Experiences, Outdoor Play, and Long-term Adiposity Changes Among Preschool- and School-Aged Children in Singapore 1 Year After Lockdown

**DOI:** 10.1001/jamapediatrics.2021.5585

**Published:** 2022-01-24

**Authors:** Ka Kei Sum, Shirong Cai, Evelyn Law, Bobby Cheon, Geoffrey Tan, Evelyn Loo, Yung Seng Lee, Fabian Yap, Jerry Kok Yen Chan, Mary Daniel, Yap Seng Chong, Michael Meaney, Johan Eriksson, Jonathan Huang

**Affiliations:** 1 Singapore Institute for Clinical Sciences, Agency for Science, Technology and Research, Singapore; 2 Department of Paediatrics, Yong Loo Lin School of Medicine, National University Singapore, Singapore; 3 School of Social Sciences, Nanyang Technological University, Singapore; 4 Institute of Mental Health, Singapore; 5 KK Women’s and Children’s Hospital, Singapore; 6 Department of Obstetrics & Gynaecology, Yong Loo Lin School of Medicine, National University Singapore, Singapore

## Abstract

**Importance:**

Despite the potential for COVID-19 infection control–related events to have an effect on child well-being, comprehensive assessments of postlockdown changes and persistent outcomes are lacking.

**Objective:**

To survey the extent of COVID-19 lockdown–related lifestyle changes, their differences by child age and family socioeconomic status, and the potential association with child adiposity 1 year after lockdown.

**Design, Setting, and Participants:**

A self-administered, electronic survey was introduced to 2 ongoing child cohorts (the Singapore Preconception Study of Long-term Maternal and Child Outcomes [S-PRESTO] cohort of preschool children aged 1-4.5 years and the Growing Up in Singapore Towards Healthy Outcomes [GUSTO] cohort of primary school children aged 9-10.7 years) from July 8, 2020, to September 5, 2020, which was 1 to 3 months after the end of strict universal movement restrictions (duration of 73 days ending on June 19, 2020). All active participants from S-PRESTO and GUSTO, 2 population-based, longitudinal, parent-offspring cohorts in Singapore, were invited to participate and monitored through June 15, 2021.

**Exposures:**

Exposures included family income before and after the COVID-19 lockdown, changes in child outdoor play or exercise, and COVID-19–related life events; all data were self-reported by parents and school-aged children 1 to 3 months after the lockdown.

**Main Outcomes and Measures:**

Primary outcomes were self-reported COVID-19–related life events and changes in child online socialization, outdoor play or exercise, and intrafamily relationships. Study staff measured children’s weight, height, and skinfold thickness before and up to 1 year after lockdown. Body mass indices (calculated as weight in kilograms divided by height in meters squared) and World Health Organization–standardized scores were computed. Differences before and after lockdown were compared using baseline-adjusted linear regression.

**Results:**

A total of 604 parents (53% of active cohort participants) and 356 school-aged children completed the survey and were similar to source cohorts. This represents 373 of 761 eligible children in the GUSTO cohort (mean [SD] age, 9.9 [0.4] years; 197 girls [52.8%]) and 231 of 370 eligible children in the S-PRESTO cohort (mean [SD] age, 2.6 [0.8] years; 121 boys [52.6%]). The COVID-19–related life changes were prevalent and varied (eg, 414 of 600 children [69.0%] reported changes in social activities). More than one-third of primary school–aged children (122 of 356 [34.3%]) and one-quarter of preschool-aged children (56 of 229 [24.5%]) eliminated any outdoor play after the lockdown. Lower family income before the lockdown was associated with increased odds of elimination of outdoor play (adjusted odds ratio per 1000 Singapore dollars [$730 US dollars] decrease, 1.09; 95% CI, 1.01-1.19). Complete elimination of outdoor activity (vs continued outdoor activity) was associated with an increase in body mass index of 0.48 (95% CI, 0.03-0.94) and a body mass index *z* score of 0.18 units (95% CI, 0-0.37) in school-aged children approximately 1 year after lockdown.

**Conclusions and Relevance:**

Results of this cohort study suggest that 1 to 3 months after a brief, strict lockdown, a large proportion of parents and school-aged children reported elimination of outdoor play, which was more prevalent in lower-income families. Future research to better understand clinical implications and ways to mitigate lockdown outcomes is essential.

## Key Points

**Question:**

What are typical lifestyle changes experienced by children after COVID-19–related lockdowns, and what are potential long-term outcomes?

**Findings:**

In this cohort study of 604 children, one-third of parents and school-aged children reported elimination of outdoor play or exercise, and those with lower family income before and after lockdown were more likely to report elimination of outdoor play. Elimination of play was associated with increased adiposity 1 year after lockdown in school-aged children but not preschool-aged children.

**Meaning:**

Outdoor play is an important part of children’s well-being, and efforts to mitigate avoidable negative outcomes of COVID-19 pandemic–related lockdowns should be considered.

## Teaser

This cohort study investigates the life experiences, outdoor activity level, and adiposity changes in children in Singapore 1 year after COVID-19–related lockdown.

## Introduction

The COVID-19 pandemic has led to concern about the potential outcomes of school closures and movement restrictions on the health and well-being of children.^1,2,3,4,5,6^ Although there is growing evidence of changes in child behaviors, such as sedentary behavior or screen time, physical activity, diet, and sleeping duration, during lockdown in several countries,^4,5,6,7,8,9,10,11,12,13,14,15,16^ given the adoption of a diverse spectrum of policies across the globe, there has been little systematic evidence on the persistence of effects (or the spectrum of COVID-19–related life events that may cause them) after strict COVID-19–related control measures have ended. This is particularly true for regions where lockdown measures were stringent and effective and where daily activities, such as schooling and extracurricular activities, have largely resumed. It is unclear whether postulated harms, such as deteriorating familial relationships or acquisition of maladaptive behaviors (eg, inactivity,^4,5,6,7,8,9,10,11,12,13,14,15,16^ increased leisure screen time,^7,8,9,10,11,12,14,15,16^ or change in sleep patterns^7,8,14^), continue to persist after lockdown. Early studies have shown that these behavior changes during lockdown may continue after lockdown among youths in China^17^ and primary school children in Cyprus.^18^ Although it has been speculated that such changes may exacerbate trends in childhood obesity,^19,20^ studies thus far have only shown changes in child adiposity immediately after lockdown.^17,21,22^ There are no studies, to our knowledge, of longer-term consequences and whether child outcomes may differ by developmental stage or family sociodemographic characteristics.

Drawing from 2 population-based, longitudinal child cohorts (the Singapore Preconception Study of Long-term Maternal and Child Outcomes [S-PRESTO] cohort^23^ of preschool children aged 1-4.5 years and the Growing Up in Singapore Towards Healthy Outcomes [GUSTO] cohort^24^ of primary school children aged 9-10.7 years), we described the extent to which families experienced significant COVID-19–related life events, family dynamics, and changes in child behaviors after lockdown in a setting where mandatory movement restrictions were strict, but brief, and followed by resumption of schools and most other daily activities. On the other hand, as most Singaporean residents live in dense multifamily buildings with shared recreational facilities, behavior changes may be larger and more durable.^5^ We investigated whether changes in child behaviors were associated with child age group, family income, and COVID-19–related life events. Finally, leveraging continued follow-up in our cohorts, we investigated whether changes in lockdown-related behaviors were associated with changes in objective adiposity measures 1 year after lockdown.

## Methods

### Population and Data Collection

In Singapore, movement control orders began on April 7, 2020, as part of circuit-breaker measures to slow the spread of SARS-CoV-2 and included near-universal workplace, school, and public space closures with homestay mandates (except for essential activities, eg, grocery shopping, exercise, work for essential services). Many measures ended June 1, 2020, including gradual reopening of schools, with broader reopening of public facilities, such as playgrounds, beginning on June 19, 2020, for a total of 73 days of more stringent measures. From July 8 to September 5, 2020, approximately 1 to 3 months after the cessation of Singapore’s movement control restrictions, we invited parents and child participants of the 2 population-based prospective child cohorts S-PRESTO^23^ and GUSTO^24^ to complete self-administered electronic surveys (REDCap; Vanderbilt University). These surveys allowed parents and children to document their COVID-19–related experiences. This research was approved by the National University of Singapore Institutional Review Board, SingHealth Centralized Review Board, and National Healthcare Group Domain Specific Review Board (Singapore). Written informed consent was obtained from all parents (with assent from primary school–aged children). This study followed the Strengthening the Reporting of Observational Studies in Epidemiology (STROBE) reporting guidelines.

Parents were administered a modified Life Experiences Survey^25^ where they were asked to indicate whether they had experienced any of approximately 60 events in the past year. Parents were asked to indicate whether the event was extremely negative (−3) to extremely positive (3), whether the event occurred within the past 6 months or 6 to 12 months prior, and whether they believed the event was COVID-19 related. Parents were also asked about COVID-19–related changes in income, experiences of illness, quarantine, the sleeping patterns of their child, their relationship with their child, and changes in the usual activities of their child, including playing with friends, online socialization (social media) activity, video chatting with friends and family, and outdoor play or exercise, before April 7, 2020 (start of the circuit-breaker measures), and in the month preceding the completion of the questionnaire (July 8 to September 5, 2020). Particularly, we defined outdoor play elimination as parent report of any nonzero frequency of outdoor play before lockdown and then no outdoor play after lockdown, excluding those cases where parents consistently reported no outdoor play both before and after lockdown (33 of 356 [9.3%] in the GUSTO cohort; 4 of 229 [1.7%] in the S-PRESTO cohort). Children in the GUSTO cohort were asked separately to complete similar questions. These data were merged with sociodemographic data from all previous waves of the 2 cohorts. Original S-PRESTO inclusion criteria included women younger than 35 years, those who were not currently pregnant, those with a plan to conceive within 1 year after recruitment, and residency in Singapore for 5 years.^23^ Women with a history of infertility or diabetes, those taking corticosteroids or anticonvulsants, and those taking medications for fertility, HIV, or hepatitis were excluded. Original GUSTO inclusion criteria included women 18 years or older, those with residency in Singapore for at least 5 years; those with an intent to deliver in Singapore; those who self-reported Chinese, Indian, or Malay ancestry; and those who had their first trimester ultrasound examinations at 1 of 2 major public maternity wards in Singapore.^24^ Singapore follows a formal racial-ethnic model where citizens are classified as Chinese, Malay, Indian, or Other for various policy and representational purposes. Self-reported child ancestry from a single ethnicity (maternal and paternal) was an inclusion criterion for GUSTO but not S-PRESTO. Because this a priori classification has both historical and current environmental and policy relevance, they were collected and reported in both cohorts. Women pregnant with a fetus older than 14 gestational weeks, those currently receiving chemotherapy or psychotropic medications, and those with a history of type 1 diabetes at recruitment were excluded. All participants who completed S-PRESTO or GUSTO study visits before lockdown (at delivery or at year 8, respectively) were considered the referent source population.

Because differential fat deposition may have different effects on cardiometabolic health not captured by body mass index (BMI; calculated as weight in kilograms divided by height in meters squared) alone, we extracted all study staff–measured anthropometrics (ie, weight, height, skinfold thickness, and abdominal circumference) available from both of the most recent completed study waves before and after lockdown (through June 2021) along with date and age at collection. For adiposity change comparisons, we limited models to participants with prelockdown measurements within 1 year of the start of lockdown. All BMI *z* scores were calculated using the World Health Organization child growth standards. To account for right skew, skinfold thicknesses were natural log transformed for analyses.

### Statistical Analysis

We examined participants’ family sociodemographic characteristics and survey responses using standard descriptive statistics (Table) and plots. Differences in variable distributions before vs after lockdown were tested using paired *t* tests for continuous variables and paired Wilcoxon rank sum tests for ordinal variables. We explored whether the COVID-19–related life events reported using the Life Experiences Survey differed by socioeconomic position. We regressed the binary report of each specific life event (yes/no) and ordinal rating (−3 to 3, for those experiencing that event) on household income and cohort membership using logistic regression and ordinal logistic regression, respectively. Only events with more than 20 reports were included in these analyses to avoid the issue of sparsity, resulting in 28 events being tested. To account for multiple testing, we used a Wald test *P* value < .001 (.05/28 life events) as indicators of potential differences, and corresponding 99.9% CIs were presented along with point estimates.

**Table.** Sociodemographic Characteristics of Participants, Overall and by Cohort

Variable	No./total No. (%)		
	Overall (N = 604)	Primary school–aged cohort (GUSTO; n = 373)	Preschool-aged cohort (S-PRESTO; n = 231)
Respondent			
Father	12/604 (2.0)	11/373 (3.0)	1/231 (0.4)
Mother	592/604 (98.0)	362/373 (97.1)	230/231 (99.6)
Child’s sex			
Male	297/603 (49.3)	176/373 (47.2)	121/230 (52.6)
Female	306/603 (50.8)	197/373 (52.8)	109/230 (47.4)
Child’s age, mean (SD), y	7.10 (3.6)	9.87 (0.4)	2.63 (0.8)
Maternal education			
None/primary	13/599 (2.2)	13/368 (3.5)	0/231
Secondary/ITE	139/599 (23.2)	126/368 (34.2)	13/231 (5.6)
A-level/polytechnic/diploma	136/599 (22.7)	88/368 (23.9)	48/231 (20.8)
University/postgraduate	311/599 (51.9)	141/368 (38.3)	170/231 (73.6)
Ethnicity			
Chinese	402/604 (66.6)	222/373 (59.5)	180/231 (77.9)
Indian	70/604 (11.6)	58/373 (15.6)	12/231 (5.2)
Malay	124/604 (20.5)	92/373 (24.7)	32/231 (13.9)
Other^a^	8/604 (1.3)	1/373 (0.3)	7/231 (3.0)
No. of children living in the same household (other than index child)			
Median (IQR)	1 (0-2)	1 (1-2)	0 (0-1)
Total No.	551	345	206
No. of additional persons in the household^b^			
Median (IQR)	3 (2-4)	3 (2-4)	2 (1-4)
Total No.	551	345	206
Monthly household income before February 2020, SGD^c^			
0-1000	9/520 (1.7)	7/312 (2.2)	2/208 (1.0)
1001-2000	36/520 (6.9)	31/312 (9.9)	5/208 (2.4)
2001-3000	61/520 (11.7)	53/312 (17.0)	8/208 (3.9)
3001-4000	58/520 (11.2)	44/312 (14.1)	14/208 (6.7)
4001-5000	40/520 (7.7)	28/312 (9.0)	12/208 (5.8)
5001-6000	49/520 (9.4)	26/312 (8.3)	23/208 (11.1)
6001-7000	30/520 (5.8)	11/312 (3.5)	19/208 (9.1)
7001-8000	43/520 (8.3)	19/312 (6.1)	24/208 (11.5)
8001-9000	28/520 (5.4)	17/312 (5.5)	11/208 (5.3)
9001-10 000	34/520 (6.5)	14/312 (4.5)	20/208 (9.6)
>10 000	132/520 (25.4)	62/312 (19.9)	70/208 (33.7)
Monthly household income from March-June 2020, SGD^c^			
0-1000	23/522 (4.4)	17/317 (5.4)	6/205 (2.9)
1001-2000	47/522 (9.0)	41/317 (12.9)	6/205 (2.9)
2001-3000	67/522 (12.8)	55/317 (17.4)	12/205 (5.9)
3001-4000	56/522 (10.7)	41/317 (12.9)	15/205 (7.3)
4001-5000	38/522 (7.3)	23/317 (7.3)	15/205 (7.3)
5001-6000	46/522 (8.8)	30/317 (9.5)	16/205 (7.8)
6001-7000	29/522 (5.6)	10/317 (3.2)	19/205 (9.3)
7001-8000	39/522 (7.5)	15/317 (4.7)	24/205 (11.7)
8001-9000	31/522 (5.9)	20/317 (6.3)	11/205 (5.4)
9001-10 000	29/522 (5.6)	12/317 (3.8)	17/205 (8.3)
>10 000	117/522 (22.4)	53/317 (16.7)	64/205 (31.2)
Income decreased from before February to March-June 2020^c^			
Families with decreased income	124/515 (24.1)	76/310 (24.5)	48/205 (23.4)

Abbreviations: GUSTO, Growing Up in Singapore Towards Healthy Outcomes; S-PRESTO; Singapore Preconception Study of Long-term Maternal and Child Outcomes; SGD, Singapore dollars.

^a^ Other is an official Singaporean racial-ethnic category and includes mothers who self-reported either mixed ancestry or ethnicity other than Chinese, Indian, or Malay.

^b^ Excluding respondent and child.

^c^ In SGD (1 SGD = $0.73 US dollars).

Next, we examined factors associated with elimination of all outdoor play and exercise (any before lockdown and none in the month before completing the survey). In logistic regression models, we modeled odds of elimination using household income before lockdown, household income after lockdown, and experience of the 10 most common life events (from the Life Experiences Survey). Each model was adjusted for cohort membership, maternal self-reported ethnicity, and maternal education. For income, quadratic terms were included, and evidence of nonlinear contribution was assessed by likelihood ratio test. Finally, we examined differences in long-term changes in continuous adiposity measures using paired *t* tests (prelockdown and 1-year postlockdown measures) stratified by cohort membership. We estimated the association between outdoor play elimination and postlockdown adiposity measures by adjusting for corresponding prelockdown measure and precision variables: age, sex, time elapsed since end of lockdown, and an elimination × cohort interaction term (base model). Finding no associations in the preschool-aged cohort, we focused on the primary school–aged cohort and fit final models adjusted for conventional confounders (a priori associated with the child adiposity outcome and associated with elimination of outdoor play in this sample): maternal education, ethnicity, and prelockdown household income. Modeling assumptions were evaluated by examining residual plots. All regression analyses were conducted on a complete case basis, and *P* values and 95% CIs were presented for multivariable adjusted models taking a *P* value < .05 as a study-specific threshold for significance. Analyses were conducted using R, version 3.6.2 (R Foundation); R packages *base, tidyverse*, *ggplot2*, and *gtsummary*, version 1.2.1335 (RStudio); and Stata, version 16.1 (StataCorp).

## Results

**Figure 1.**
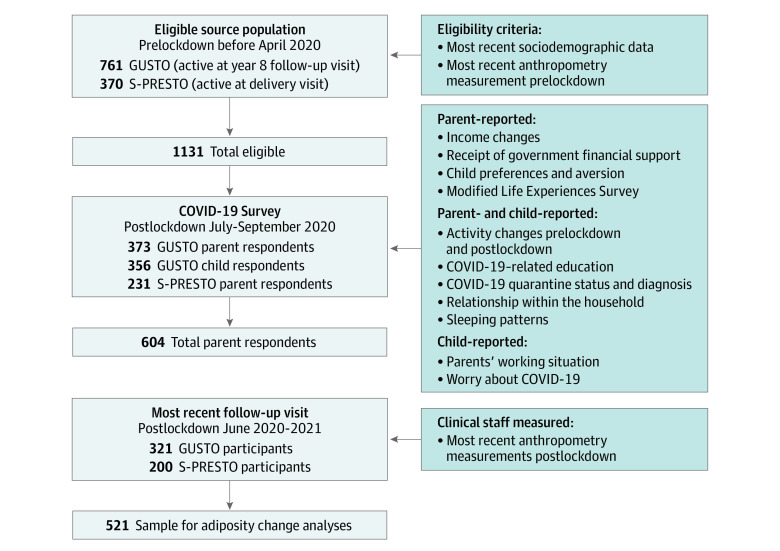
Flowchart of Eligible Source Population and COVID-19 Life Experiences Data Collection

We received responses from 604 parents (53% of active cohort participants). There were 373 of 761 eligible children in the GUSTO cohort (mean [SD] age, 9.9 [0.4] years; 197 girls [52.8%]; 176 boys [47.2%]) and 231 of 370 eligible children in the S-PRESTO cohort (mean [SD] age, 2.6 [0.8] years; 109 girls [47.4%]; 121 boys [52.6%]) (Table; eTable 1 in the Supplement). A total of 356 of 373 primary school–aged children (95.4%) completed the survey (Figure 1). Among GUSTO participants, 222 of 373 (59.5%) had previously self-reported maternal ethnicity as Chinese, 58 (15.5%) as Indian, 92 (24.7%) as Malay, and 1 (0.3%) as Other (in the Singaporean system, Other indicates mixed ancestry or an ethnicity other than Chinese, Malay, or Indian). Among S-PRESTO participants, 180 of 231 (77.9%) had previously self-reported maternal ethnicity as Chinese, 12 (5.2%) as Indian, 32 (13.9%) as Malay, and 7 (3.0%) as Other. Respondents to the survey were generally representative, with the exception that mothers in the GUSTO cohort had slightly higher education than their source cohort (eTable 1 in the Supplement). Compared with mothers from the primary school–aged cohort (GUSTO), mothers from the preschool cohort (S-PRESTO) were more likely to be university graduates (170 of 231 [73.6%] vs 141 of 368 [38.3%]), self-identify as Chinese (180 of 231 [77.9%] vs 222 of 373 [59.5%]), have higher median (IQR) household income before and after lockdown (S-PRESTO median, SGD 7001-8000 [US $5111-$5840]; IQR, SGD 5001-6000 [US $3651-$4380] to SGD 10 000 and higher [US $7300 and higher] vs GUSTO median, SGD 4001-5000 [US $2921-$3650]; IQR, SGD 2001-3000 [US $1461-$2190] to SGD 8001-9000 [US $5841-$6570]), and have fewer median (IQR) household members (2 [1-4] members vs 3 [2-4] members) (Table). Parents from both cohorts were equally likely to report decreases in household income (48 of 205 [23.4%] vs 76 of 310 [24.5%]) (Table).

### Child Experiences and Behavior Change

**Figure 2.**
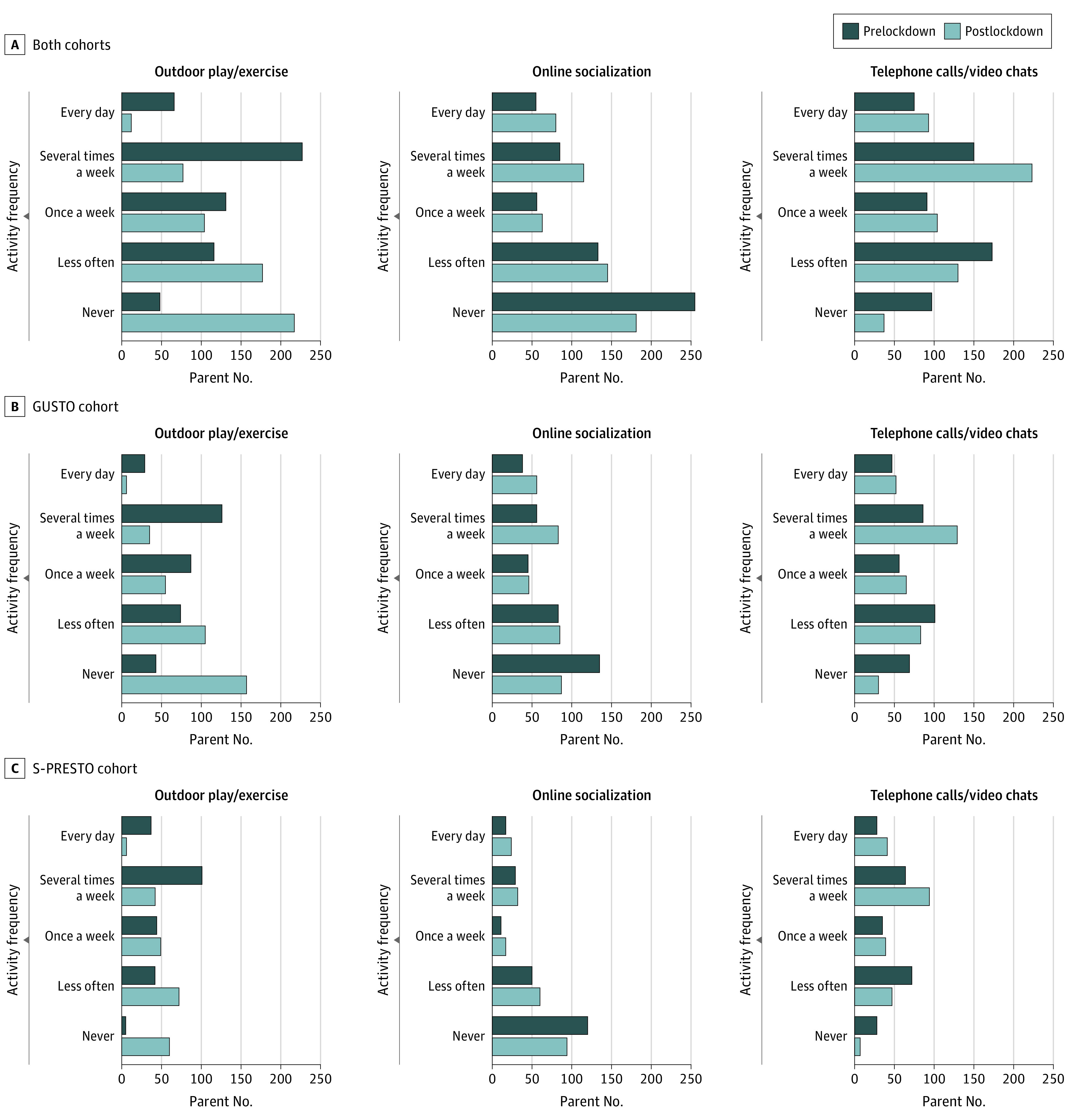
Changes in Frequency Distributions of Activities From Before to After Lockdown Paired Wilcoxon tests of frequency differences before and after lockdown were all significant (*P* < .001) for (A) both cohorts combined, (B) the Growing Up in Singapore Towards Healthy Outcomes (GUSTO) cohort, and (C) the Singapore Preconception Study of Long-term Maternal and Child Outcomes (S-PRESTO) cohort.

A large proportion of parents reported significant decreases in outdoor play or exercise (375 of 585 [64.1%]; paired Wilcoxon test, *P* < .001) and increases in online socialization (eg, social media or online games, 177 of 584 [30.3%], paired Wilcoxon test, *P* < .001) and telephone or video calls (220 of 586 [37.5%]; paired Wilcoxon test, *P* < .001) with friends and family after lockdown (Figure 2; eFigure 1 in the Supplement). Compared with primary school–aged children, preschool-aged children were more likely to increase telephone or video calls (97 of 227 [42.7%] vs 123 of 359 [34.3%]) and less likely to eliminate all outdoor play or exercise (56 of 229 [24.5%] vs 122 of 356 [34.3%]) (eFigure 1 in the Supplement). Parents from both cohorts and primary school–aged children in the GUSTO cohort widely reported learning about COVID-19–related topics, particularly handwashing (441 of 489 parents [90.2%]; 320 of 355 primary school–aged children [90.1%]), mask wearing (428 of 489 parents [87.5%]; 288 of 355 primary school–aged children [81.1%]), and social distancing (393 of 489 parents [80.4%]; 271 of 355 primary school–aged children [76.3%]).

Strategies to manage feelings and emotions were least reported (227 of 489 parents [46.4%]; 181 of 355 primary school–aged children [51.0%]) (eFigure 2 in the Supplement). Parents from both cohorts and primary school–aged children were more likely to report improved familial relationships: 308 of 591 parents (52.1%) reported slightly or greatly improved relationships, and only 20 of 591 (3.4%) reported worsening relationships (eFigure 3 in the Supplement). Children reported less than 10 hours of sleep per night during lockdown, even on weekends or public holidays (eFigure 4 in the Supplement). Few parents reported children who had some aversions to seeing friends (35 of 591 [5.9%]), playing outdoors (83 of 591 [14.0%]), or going shopping or dining out (98 of 591 [16.6%]) (eFigure 5 in the Supplement).

### Diverging Experiences of Major Life Changes

Based on the administered Life Experiences Survey (eAppendix in the Supplement), substantial proportions of families reported major COVID-19–related life changes in social activities (414 of 600 [69.0%]), work situations (330 of 597 [55.3%]), recreation (307 of 597 [51.4%]), schooling (233 of 596 [39.1%]), and financial status (171 of 599 [28.6%]) (eTable 2 and eFigure 6 in the Supplement). Common events were given highly divergent scores (many rated both positively and negatively), such as changes in work situation (151 positive vs 159 negative) and major changes in eating habits (74 positive vs 82 negative) (eFigure 7 in the Supplement). Higher prelockdown household income was associated with greater likelihood of changes in work situation but lesser likelihood of reporting major changes in financial status (adjusted odds ratio [aOR] per SGD 1000 [US $730] higher income, 0.90; 99.9% CI, 0.81-1.00) (eFigure 8 in the Supplement). Possibly owing to the diverging ratings, income was not associated with event ratings except for a more negative rating for changes in social activities (12% lower odds of a higher rating per approximately SGD 1000 [US $730] higher income; aOR, 0.88; 99.9% CI, 0.79-0.99) (eFigure 9 in the Supplement).

### Associations With Outdoor Play Elimination

**Figure 3.**
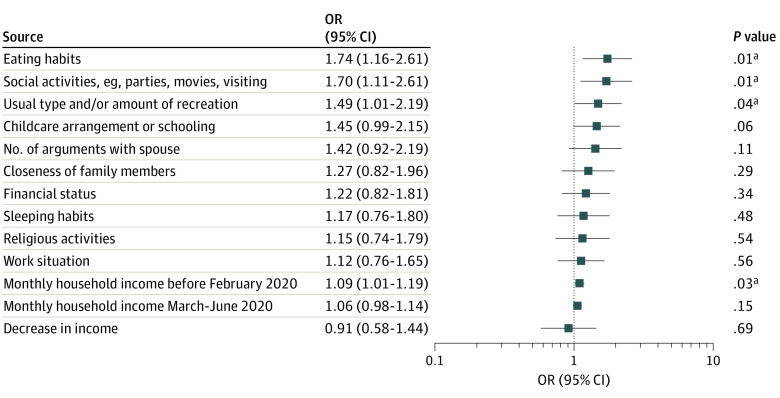
Association Between Risk Factors and Odds of Eliminating All Outdoor Play or Exercise After Lockdown Logistic regression adjusted for cohort membership, maternal self-reported ethnicity, and maternal education. Monthly household income rows represent per SGD 1000 (US $730) decrease. ^a^*P* < .05.

Lower household incomes, either before or after lockdown, were associated with increased odds of reporting the elimination of all outdoor play or exercise (aOR per SGD 1000 [US $730] lower prelockdown income, 1.09; 95% CI, 1.01-1.19; *P* = .03). Major changes in eating habits, social activities, and amount of recreation were also associated with elimination of outdoor play (Figure 3; eTable 3 in the Supplement).

### Adiposity Measures Before and After Lockdown

**Figure 4.**
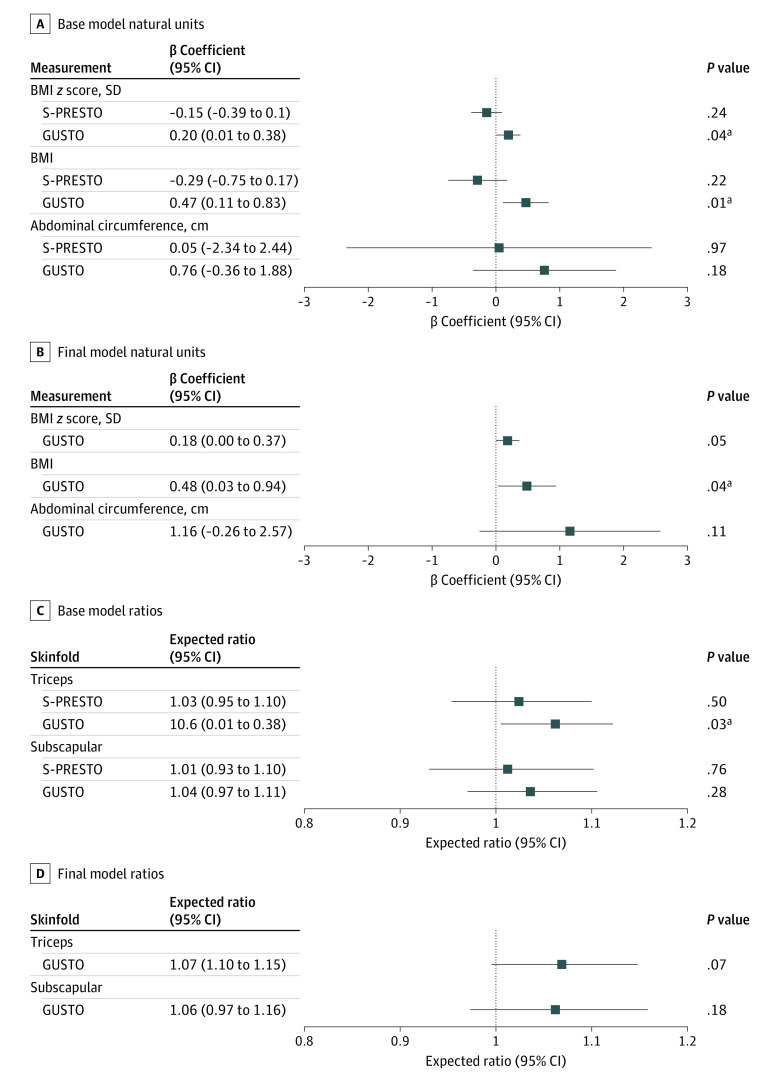
Adjusted Associations Between Eliminating Outdoor Play and Adiposity Measures After Lockdown Among Those Who Reported Any Outdoor Play Before Lockdown Base models (A, point estimates in natural units; C, in ratios) were adjusted for baseline prelockdown measures and precision variables (age, sex, and days since end of lockdown) and interacted with cohort (*P* values for interaction = .01 for BMI and .03 for BMI *z* score). Final models (B, point estimates in natural units; D, in ratios) were further adjusted for confounders: ethnicity, maternal education, and prelockdown household income. Final models were fit for primary school–aged children (Growing Up in Singapore Towards Healthy Outcomes [GUSTO]) only because of null crude association in the preschool-aged children. Point estimates are provided in natural units and ratios of eliminated to not eliminated (due to log-transformation); 95% CIs and *P* values are reported. ^a^*P* < .05.

Among primary school–aged children, all standardized and unstandardized adiposity measures were higher after lockdown than before lockdown (eTable 4 in the Supplement). In primary school–aged children, mean (SD) BMI *z* score after lockdown was 0.57 (1.50) or 0.27 SDs higher than prelockdown values (0.30 [1.50]; *P* < .001). In preschool-aged children, mean (SD) BMI *z* score after lockdown was 0.07 (1.03) or only 0.04 SDs higher (*P* = .52) than before lockdown (0.03 [1.00]). In preschool-aged children, after lockdown, triceps skinfold *z* score (0.17; 95% CI, 0.05-0.30; *P* = .006) but not subscapular *z* score (−0.02; 95% CI, −0.16 to 0.12; *P* = .76) was increased compared with prelockdown values (mean triceps skinfold *z* score [SD], 0.79 [0.97]; subscapular *z* score, 0.43 [0.99]). In base change models, elimination of outdoor play or exercise was associated with increased BMI (0.47; 95% CI, 0.11-0.83; *P *= .01), BMI *z* score (0.2; 95% CI, 0.01-0.38; *P *= .04) and triceps skinfold thickness in primary school–aged children only (1.06; 95% CI, 1.01-1.12; *P* = .03) (Figure 4). These associations mostly persisted in final models adjusted for sociodemographic characteristics, including BMI (0.48; 95% CI, 0.03-0.94; *P *= .04) and BMI *z* score (0.18; 95% CI, 0-0.37; *P *= .05) for elimination of outdoor play vs no elimination of outdoor play.

## Discussion

In this cohort study, we found many anticipated lockdown-related changes in recreation, working arrangements, and child schooling. Responses (positive or negative) to such changes were often highly divergent. As expected, online socialization and video chats or calls with friends or family increased. A substantial proportion of participants reported no outdoor play or exercise after lockdown, and this was associated with an increase in adiposity 1 year onward even adjusted for baseline adiposity. Furthermore, we found that those families with lower prelockdown household income were more likely to report substantial changes in financial status and elimination of outdoor play or exercise for their children.

Studies of long-term postlockdown follow-up are only beginning to emerge. Although findings from Canada^16^ showed reduced outdoor play during lockdown, our study results suggest that this outcome persisted beyond the lockdown period. In contrast, studies from the US,^13,14^ Germany,^15^ and the Netherlands^10^ showed an increase in outdoor play for children during the lockdown period. This may be explained by between-country differences in policies and typical housing or neighborhood composition.^5^ In Singapore, where most people live in high-density apartment blocks with shared facilities and playgrounds that were closed during lockdown, reductions in child play were expected^26^ during the lockdown (not after the facilities were reopened). Public parks remained mostly open for exercise (eg, jogging, cycling) throughout the lockdown period.

Ceasing outdoor play or exercise was more frequently reported by parents of older children than by those with younger children in our study, which is consistent with previous findings.^11,14^ The complete cessation of such activity may be related to the typical outdoor activities engaged by primary school–aged children, such as organized sports, which still faced restrictions after lockdown. Preschool-aged children take part in more unstructured free play, which may occur in unrestricted spaces. This finding is supported by observations in the US^14^ and Germany^15^ where unstructured play was more common in younger children during lockdown, and there were less changes in physical activity in younger children overall.^13,16^ Parents with lower prelockdown household income were more likely to report no outdoor play or exercise after lockdown. A Spanish study^12^ also demonstrated that lifestyle deterioration during lockdown, such as reduction in physical activity, was more prevalent in children from families with social vulnerabilities.

To our knowledge, there are no studies to date that have specifically studied if elimination of outdoor play or exercise results in adiposity changes in children after lockdown. This is important because it is highly likely that active play indoors may not compensate for reduced outdoor play.^16^ Our study results showed that all standardized adiposity measures in primary school–aged children were observed to be consistently higher in the postlockdown period than during the prelockdown period. In our cohort, the mean BMI increase of 1.5 found in primary school children is consistent with a US study^21^ of children aged 6 to 11 years where a 1.11 increase in BMI was observed when compared 3 months before and after lockdown. Moreover, we found both BMI (0.48) and BMI *z* score (0.18) values were substantially higher in primary school–aged children who specifically reported the elimination of outdoor play. In contrast, studies in Israel^22^ and the US^21^ 2 to 3 months after lockdown found that increases in weight percentiles and obesity prevalence tend to be more significant in children younger than 6 years. In our study of preschool-aged children, 1 year after lockdown, we only found nonsignificant increases in BMI *z* score. Additionally, elimination of outdoor play showed small nonsignificant decreases in BMI and BMI *z* scores. This may reflect differences in age and length of follow-up between studies. Younger, smaller children may get sufficiently vigorous activity though only indoor play, such as by playing on household furniture. Nonetheless, our findings reflect the importance of examining intermediate outcomes of lockdown. Previous work in our population,^27^ as elsewhere, suggests that the age of 2 years is a critical time in a child’s life for the establishment of future adiposity and metabolic risk. The results of this study showed an inverse association between changes in outdoor play after the COVID-19 lockdown and increased adiposity in preschool-aged children. Therefore, it is possible that lockdown-related changes in outdoor play may not be associated with future metabolic risk in these preschool-aged children.

### Strengths and Limitations

This study described a wide range of exposures and behavioral changes in families after the COVID-19 lockdown and a return to many prelockdown activities. We assessed objective measures of long-term adiposity outcomes. Furthermore, this study was nested within 2 distinct population-based longitudinal cohorts which allowed us to not only compare experiences at different ages but also to examine many predictors and potential mediators of lockdown-related changes.

This study has some limitations, including the lack of precision in some measurements (eg, the retrospective self-reporting of physical activity and eating behaviors) owing to the necessary brevity of the self-administered online survey format. More precise measures (eg, accelerometry) would have been preferable but were not collected during a relevant window. However, the nested design may allow future studies to take advantage of ongoing data collection including actigraphy and food diaries. Our results may only be applicable to this particular urban context. Only 2% of respondents lived in landed properties (ie, detached, single-family dwellings), and we were not able to directly investigate other contextual variables, such as living space or neighborhood amenities. Many outstanding questions, such as the role of sleep disruption and dietary changes, were outside the scope of this initial study and should be investigated with more focused analyses and data from ongoing follow-up of the cohorts.

## Conclusions

In this cohort study, results suggest that lower prelockdown socioeconomic status was associated with greater changes in physical activity and long-term adiposity than higher socioeconomic status. Although both parents and children reported improved familial relationships and increases in socialization via digital means after the lockdown, a large percentage of children reported no outdoor play or physical activity 1 to 3 months after the lockdown. Our results suggest that this activity cessation was associated with increased adiposity in primary school–aged children 1 year after lockdown. Our results further suggest that children’s return to normal prelockdown activity levels should not be taken for granted, and interventions to ensure that adequate postlockdown physical activity resumes may be warranted. Follow-up for behavioral and clinically relevant outcomes will continue to be essential because the reduction in outdoor physical activity may exacerbate childhood obesity if the patterns remain over the long term.

## Notes

Supplement. **eTable 1. **Characteristics of Respondents Versus Nonrespondents **eTable 2. **Top Ten Most Commonly Reported Major Life Experiences in the Past Six Months **eTable 3. **Risk Factors to Ceasing Outdoor Play or Exercise **eTable 4. **Adiposity Measures Prelockdown and Postlockdown Among Children in the Study **eFigure 1. **Parent-Reported Changes in Child’s Social Activities by Cohort **eFigure 2. **Frequency of COVID-19–Related Education or Discussion **eFigure 3. **Family Dynamics Postlockdown Compared to Prelockdown Period as Reported by School-Aged Child or Parent **eFigure 4. **Parent- and Child-Reported Child Sleep Duration in the Postlockdown Period in School-Aged Children (GUSTO) Only **eFigure 5. **Parent-Reported Child Desire or Aversion to Activities Outside the Home During Lockdown **eFigure 6**. Average Ratings of the Parent-Reported Life Experiences in the Order of Most Negative to Most Positive **eFigure 7. **Non-Neutral (Positive or Negative Ratings) of Major Recent Life Experiences, in Descending Order of Negative Ratings **eFigure 8**. Association Between Prelockdown Household Income (per SGD 1000 Higher Income) and Odds of Having a Major Life Experience (yes/no), Adjusted for Cohort Membership **eFigure 9. **Association Between Prelockdown Household Income (per SGD 2000 Higher Income) and Rating of Major Life Experience Among Those Who Experienced That Event, Adjusted for Cohort Membership **eAppendix.** Modified Life Experiences Survey – COVID-19
