# Supplementary material for: COVID-19–Related Life Experiences, Outdoor Play, and Long-term Adiposity Changes Among Preschool- and School-Aged Children in Singapore 1 Year After Lockdown
Source: JAMA Pediatr. 2022 Jan 24;176(3):1–10. doi: 10.1001/jamapediatrics.2021.5585 (PMC8787686; doi:10.1001/jamapediatrics.2021.5585)
Supplement: Supplement. — eTable 1. Characteristics of Respondents Versus Nonrespondents eTable 2. Top Ten Most Commonly Reported Major Life Experiences in the Past Six Months eTable 3. Risk Factors to Ceasing Outdoor Play or Exercise eTable 4. Adiposity Measures Prelockdown and Postlockdown Among Children in the Study eFigure 1. Parent-Reported Changes in Child’s Social Activities by Cohort eFigure 2. Frequency of COVID-19–Related Education or Discussion eFigure 3. Family Dynamics Postlockdown Compared to Prelockdown Period as Reported by School-Aged Child or Parent eFigure 4. Parent- and Child-Reported Child Sleep Duration in the Postlockdown Period in School-Aged Children (GUSTO) Only eFigure 5. Parent-Reported Child Desire or Aversion to Activities Outside the Home During Lockdown eFigure 6. Average Ratings of the Parent-Reported Life Experiences in the Order of Most Negative to Most Positive eFigure 7. Non-Neutral (Positive or Negative Ratings) of Major Recent Life Experiences, in Descending Order of Negative Ratings eFigure 8. Association Between Prelockdown Household Income (per SGD 1000 Higher Income) and Odds of Having a Major Life Experience (yes/no), Adjusted for Cohort Membership eFigure 9. Association Between Prelockdown Household Income (per SGD 2000 Higher Income) and Rating of Major Life Experience Among Those Who Experienced That Event, Adjusted for Cohort Membership eAppendix. Modified Life Experiences Survey – COVID-19 [file jamapediatr-e215585-s001.pdf]

Supplementary Online Content

Sum KK, Cai S, Law E, et al. COVID-19–related life experiences, outdoor play, and long-term adiposity changes in preschool- and school-aged children in Singapore 1 year after lockdown. *JAMA Pediatr*. Published online January 24, 2022. doi:10.1001/jamapediatrics.2021.5585

eTable 1. Characteristics of Respondents Versus Nonrespondents

eTable 2. Top Ten Most Commonly Reported Major Life Experiences in the Past Six Months

eTable 3. Risk Factors to Ceasing Outdoor Play or Exercise

eTable 4. Adiposity Measures Prelockdown and Postlockdown Among Children in the Study

eFigure 1. Parent-Reported Changes in Child's Social Activities By Cohort

eFigure 2. Frequency of COVID-19–Related Education or Discussion

eFigure 3. Family Dynamics Postlockdown Compared to Prelockdown Period as Reported by School-Aged Child or Parent

eFigure 4. Parent- and Child-Reported Child Sleep Duration in the Postlockdown Period in School-Aged Children (GUSTO) Only

eFigure 5. Parent-Reported Child Desire or Aversion to Activities Outside the Home During Lockdown

eFigure 6. Average Ratings of the Parent-Reported Life Experiences in the Order of Most Negative to Most Positive

eFigure 7. Non-Neutral (Positive or Negative Ratings) of Major Recent Life Experiences, in Descending Order of Negative Ratings

eFigure 8. Association Between Prelockdown Household Income (per SGD 1000 Higher Income) and Odds of Having a Major Life Experience (yes/no), Adjusted for Cohort Membership

eFigure 9. Association Between Prelockdown Household Income (per SGD 2000 Higher Income) and Rating of Major Life Experience Among Those Who Experienced That Event, Adjusted for Cohort Membership

eAppendix. Modified Life Experiences Survey – COVID-19

This supplementary material has been provided by the authors to give readers additional information about their work.

© 2022 Sum KK et al. *JAMA Pediatrics.*

eTable 1. Characteristics of Respondents Versus Nonrespondents

	GUSTO (Primary-school-aged cohort)				Pre-school-aged cohort (S-PRESTO)			
**Variable**	**N**	**Non-respondent, N=388[1]**	**Respondent, N=373[1]**	**p-value[2]**	**N**	**Non-respondent, N=139[1]**	**Respondent, N=231[1]**	**p-value[2]**
**Ethnicity**	761			0.290	370			0.483
Chinese		210 (54.12%)	222 (59.52%)			102 (73.38%)	180 (77.92%)	
Indian		67 (17.27%)	58 (15.55%)			10 (7.19%)	12 (5.19%)	
Malay		111 (28.61%)	92 (24.66%)			19 (13.67%)	32 (13.85%)	
Others		0 (0.00%)	1 (0.27%)			8 (5.76%)	7 (3.03%)	
**Maternal age at delivery**	761	31.41 (5.15)	30.76 (5.12)	0.085	370	30.88 (3.42)	30.89 (3.10)	0.985
**Maternal education**	756			0.061	370			0.209
None/primary		27 (6.96%)	13 (3.53%)			0 (0.00%)	0 (0.00%)	
Secondary/ITE		144 (37.11%)	126 (34.24%)			15 (10.79%)	13 (5.63%)	
A-level/polytechnic/diploma		95 (24.48%)	88 (23.91%)			27 (19.42%)	48 (20.78%)	
University/postgraduate		122 (31.44%)	141 (38.32%)			97 (69.78%)	170 (73.59%)	
Missing		0	5			0	0	
**Household income**	632	5.00 (2.63)	5.01 (2.64)	0.975	295	5.94 (2.33)	6.13 (1.98)	0.464
Missing		58	71			27	48	
**Lives with partner**	733	359 (92.53%)	325 (94.20%)	0.379	334	124 (98.41%)	204 (98.08%)	>0.999
Missing		0	28			13	23	
**Total household members (excluding respondent)**	733	3.36 (1.40)	3.20 (1.48)	0.125	331	2.57 (1.43)	2.55 (1.69)	0.911
Missing		0	28			14	25	
**Accommodation type**	733			0.266	368			0.340
1 to 2-room HDB flat		10 (2.58%)	19 (5.51%)			10 (7.25%)	18 (7.83%)	
3-room HDB flat		58 (14.95%)	38 (11.01%)			23 (16.67%)	40 (17.39%)	
4 to 5-room HDB flat		257 (66.24%)	235 (68.12%)			79 (57.25%)	138 (60.00%)	
Condominium		37 (9.54%)	27 (7.83%)			11 (7.97%)	16 (6.96%)	
HUDC executive flat		10 (2.58%)	13 (3.77%)			5 (3.62%)	13 (5.65%)	
Landed property		13 (3.35%)	10 (2.90%)			6 (4.35%)	2 (0.87%)	
Others		3 (0.77%)	3 (0.87%)			4 (2.90%)	3 (1.30%)	
Missing		0	28			1	1	
**Own/rent housing**	733			0.920	368			0.864
Own		343 (88.40%)	303 (87.83%)			104 (75.36%)	179 (77.83%)	
Rent		24 (6.19%)	21 (6.09%)			12 (8.70%)	19 (8.26%)	
Occupied without paying rent		21 (5.41%)	21 (6.09%)			22 (15.94%)	32 (13.91%)	
Missing		0	28			1	1	
**Child sex**	761			0.060	369			0.452

© 2022 Sum KK et al. *JAMA Pediatrics.*

Male		210 (54.12%)	176 (47.18%)			79 (56.83%)	121 (52.61%)	
Female		178 (45.88%)	197 (52.82%)			60 (43.17%)	109 (47.39%)	
Missing		0	0			0	1	
**Child BMI (kg/m²)**	74 0	16.52 (3.22)	16.55 (2.93)	0.881				
Missing		2	19					

[1]"Non-repspondents" are all active cohort members that participated in the last completed wave prior to lockdown, but did not complete the COVID-19 survey: delivery visit (S-PRESTO) or year 8 visit (GUSTO), respectively

[2]t-test for continuous variables; Fisher's exact test for categorical variables

eTable 2. Top Ten Most Commonly Reported Major Life Experiences in the Past Six Months

Event	N	N = 604
**Q36 LES: Major change in social activities, e.g., parties, movies, visiting (increased or decreased participation)**	600	414 (69.00%)
Missing		4
**Q13 LES: Changed work situation (different work responsibility, major change in working conditions, working hours, etc.)**	597	330 (55.28%)
Missing		7
**Q29 LES: Major change in usual type and/or amount of recreation**	597	307 (51.42%)
Missing		7
**Q48 LES: Major change in childcare arrangement or schooling**	596	233 (39.09%)
Missing		8
**Q19 LES: Major change in financial status (a lot better off or a lot worse off)**	599	171 (28.55%)
Missing		5
**Q6g. Major change in eating habits (much more or much less food intake)**	595	168 (28.24%)
Missing		9
**Q4 LES: Major change in sleeping habits (much more or much less sleep)**	600	153 (25.50%)
Missing		4
**Q26 LES: Major change in number of arguments with spouse (a lot more or a lot less arguments)**	589	132 (22.41%)
Missing		15
**Q24 LES: Major change in religious activities (increased or decreased attendance)**	598	128 (21.40%)
Missing		6
**Q20 LES: Major change in closeness of family members (increased or decreased closeness)**	599	124 (20.70%)
Missing		5

Questions are adapted versions of the Life Experiences Survey (Sarason, et al. 1978) with an additional column asking whether they think the event was related to COVID-19 or subsequent control measures

© 2022 Sum KK et al. *JAMA Pediatrics.*

eTable 3. Risk Factors to Ceasing Outdoor Play or Exercise

Characteristic	N	Overall, N = 585	Ceasing outdoor play, N = 178	aOR (95%CI)[1]	p-value (Wald test)
**Monthly household income category, before Feb 2020**	514				0.03
Median (IQR)		7.00 (4.00, 11)	6.00 (4.00, 9)	1.09 (1.01, 1.19)	
Missing		71	22	22	
**Monthly household income category, Mar - Jun 2020**	516				0.15
Median (IQR)		6.00 (3.00, 10)	5.00 (3.00, 8)	1.06 (0.98, 1.14)	
Missing		69	20	20	
**Decrease in income**	509				0.69
Yes		123 (24.17%)	38 (30.89%)	0.91 (0.58, 1.44)	
No		386 (75.83%)	117 (30.31%)	1	
Missing		76	23	23	
**Received financial support through government COVID-19 relief schemes**	585				0.71
Yes		97 (16.58%)	30 (16.85%)	0.91 (0.55, 1.50)	
No		488 (83.42%)	148 (83.15%)	1	
**Major change in social activities, e.g., parties, movies, visiting**	585				0.01
Yes		409 (69.91%)	133 (32.52%)	1.71 (1.11, 2.61)	
No		176 (30.09%)	45 (25.57%)	1	
**Changed work situation**	581				0.56
Yes		326 (56.11%)	100 (30.67%)	1.12 (0.76, 1.65)	
No		255 (43.89%)	77 (30.20%)	1	
Missing		4	1		
**Major change in usual type and/or amount of recreation**	583				0.04
Yes		303 (51.97%)	100 (33.00%)	1.49 (1.01, 2.19)	
No		280 (48.03%)	77 (27.50%)	1	
Missing		2	1		
**Major change in childcare arrangement or schooling**	582				0.06
Yes		232 (39.86%)	74 (31.90%)	1.45 (0.99, 2.15)	
No		350 (60.14%)	103 (29.43%)	1	
Missing		3	1		
**Major change in financial status**	583				0.34
Yes		169 (28.99%)	60 (35.50%)	1.22 (0.82, 1.81)	

© 2022 Sum KK et al. *JAMA Pediatrics.*

No		414 (71.01%)	118 (28.50%)	1	
Missing		2	0		0.008
**Major change in eating habits**	579				
Yes		165 (28.50%)	63 (38.18%)	1.74 (1.16, 2.61)	
No		414 (71.50%)	115 (27.78%)	1	
Missing		6	0		0.48
**Major change in sleeping habits**	583				
Yes		150 (25.73%)	45 (30.00%)	1.17 (0.76, 1.80)	
No		433 (74.27%)	133 (30.72%)	1	
Missing		2	0		
**Major change in number of arguments with spouse**	574				0.11
Yes		132 (23.00%)	46 (34.85%)	1.42 (0.92, 2.19)	
No		442 (77.00%)	131 (29.64%)	1	
Missing		11	1		0.54
**Major change in religious activities**	583				
Yes		127 (21.78%)	40 (31.50%)	1.15 (0.74, 1.79)	
No		456 (78.22%)	137 (30.04%)	1	
Missing		2	1		
**Major change in closeness of family members**	584				0.29
Yes		122 (20.89%)	41 (33.61%)	1.27 (0.82, 1.96)	
No		462 (79.11%)	137 (29.65%)	1	
Missing		1	0		

[1]All models adjusted for cohort membership, maternal self-reported ethnicity, and maternal education
Income, income changes, government financial adi, and the top ten most frequently reported events were assessed

© 2022 Sum KK et al. *JAMA Pediatrics.*

eTable 4. Adiposity Measures Prelockdown and Postlockdown Among Children in the Study

Adiposity measures	N	Pre-lockdown	Post-lockdown	Difference	p-value
**BMI**					
All	508	16.81 (2.85)	17.62 (3.52)	0.81 (0.67, 1.00)	<0.001
GUSTO	319	17.25 (3.36)	18.75 (3.89)	1.5 (1.3-1.7)	<0.001
S-PRESTO	189	16.05 (1.38)	15.71 (1.39)	0.34 (0.18-0.51)	<0.001
**BMI z-score**					
All	508	0.20 (1.34)	0.39 (1.37)	0.19 (0.13-0.25)	<0.001
GUSTO	319	0.30 (1.50)	0.57 (1.50)	0.27 (0.21-0.34)	<0.001
S-PRESTO	189	0.03 (1.00)	0.07 (1.03)	0.04 (-0.08-0.16)	0.5
**Abdominal circumference (cm)**					
All	403	58.67 (12.24)	63.01(13.27)	4.3 (3.8, 4.9)	<0.001
GUSTO	317	62.59 (10.77)	67.59 (11.07)	5 (4.4, 5.6)	<0.001
S-PRESTO	86	44.25 (3.15)	46.14 (3.36)	1.9 (1.4, 2.4)	<0.001
**Biceps skinfold (mm)**					
All	349	7.98 (3.59)	9.02 (4.05)	1.0 (0.77, 1.3)	<0.001
GUSTO	318	8.14 (3.69)	9.28 (4.09)	1.1 (0.85, 1.4)	<0.001
S-PRESTO	31	6.29 (1.58)	6.39 (2.36)	0.10 (0.79, 1.0)	0.8
**Suprailiac skinfold (mm)**					
All	347	10.32 (6.96)	12.40 (7.41)	2.1 (1.7, 2.5)	<0.001
GUSTO	317	10.81 (7.01)	13.04 (7.38)	2.2 (1.8-2.7)	<0.001
S-PRESTO	30	5.23 (3.74)	5.56 (2.96)	0.33 (0.43, 1.1)	0.4
**Triceps skinfold (mm)**					
All	502	12.09 (5.03)	13.46 (5.56)	1.4 (1.1, 1.6)	<0.001
GUSTO	318	13.56 (5.62)	15.42 (6.01)	1.9 (1.5, 2.2)	<0.001
S-PRESTO	184	9.55 (2.11)	10.07 (1.95)	0.51 (0.24, 0.79)	<0.001
**Triceps z-score**					
S-PRESTO	184	0.79 (0.97)	0.97 (0.79)	0.17 (0.05, 0.30)	0.006
**Subscapular skinfold (mm)**					
All	502	9.48 (5.68)	10.28 (6.06)	0.79 (0.51, 1.1)	<0.001
GUSTO	318	10.98 (6.59)	12.34 (6.62)	1.4 (1.0, 1.8)	<0.001
S-PRESTO	184	6.90 (1.55)	6.71 (2.06)	-0.19 (-0.43, 0.05)	0.12
**Subscapular z-score**					
S-PRESTO	184	0.43 (0.99)	0.41(1.09)	-0.02 (-0.16, 0.12)	0.8

Mean differences (95% confidence intervals) and p-values from paired t-tests were reported.
Triceps and subscapular z-scores were only computed for children under 5 years, per WHO growth curve standards.

eFigure 1. Parent-Reported Changes in Child's Social Activities By Cohort

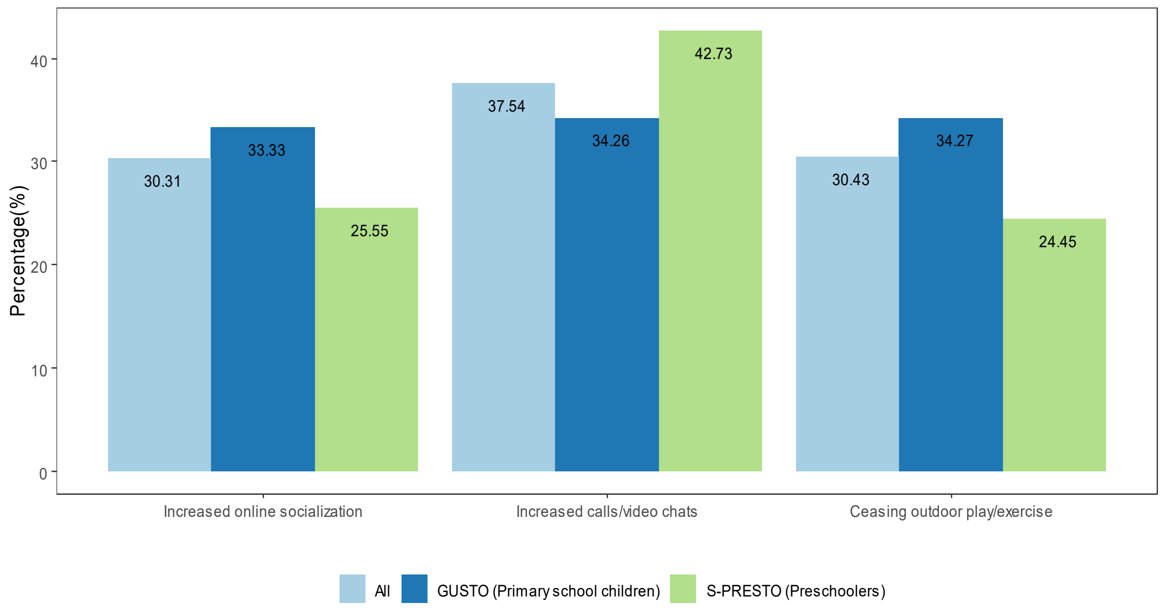

eFigure 2. Frequency of COVID-19–Related Education or Discussion

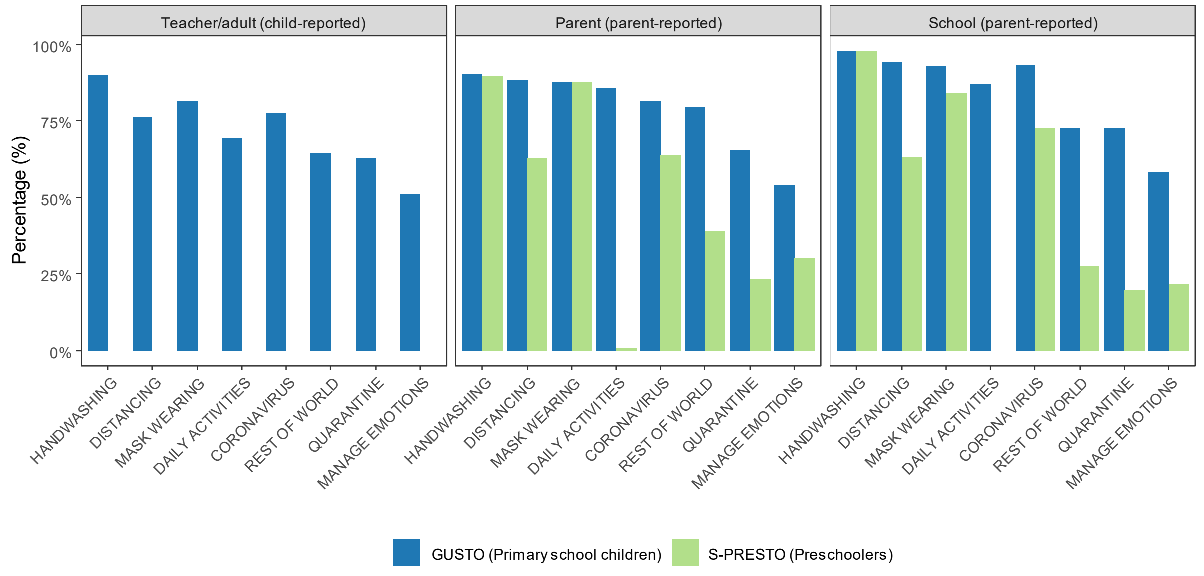

Parents were asked if children learned about a selection of COVID-19 related topics from themselves ("parent") or at school ("school"). School-aged children were asked whether they discussed any of these topics with a teacher or other adult include a parent ("teacher/adult"). The reporter, either parent or child, is given in parentheses.

© 2022 Sum KK et al. *JAMA Pediatrics.*

eFigure 3. Family Dynamics Postlockdown Compared to Prelockdown Period as Reported by School-Aged Child or Parent

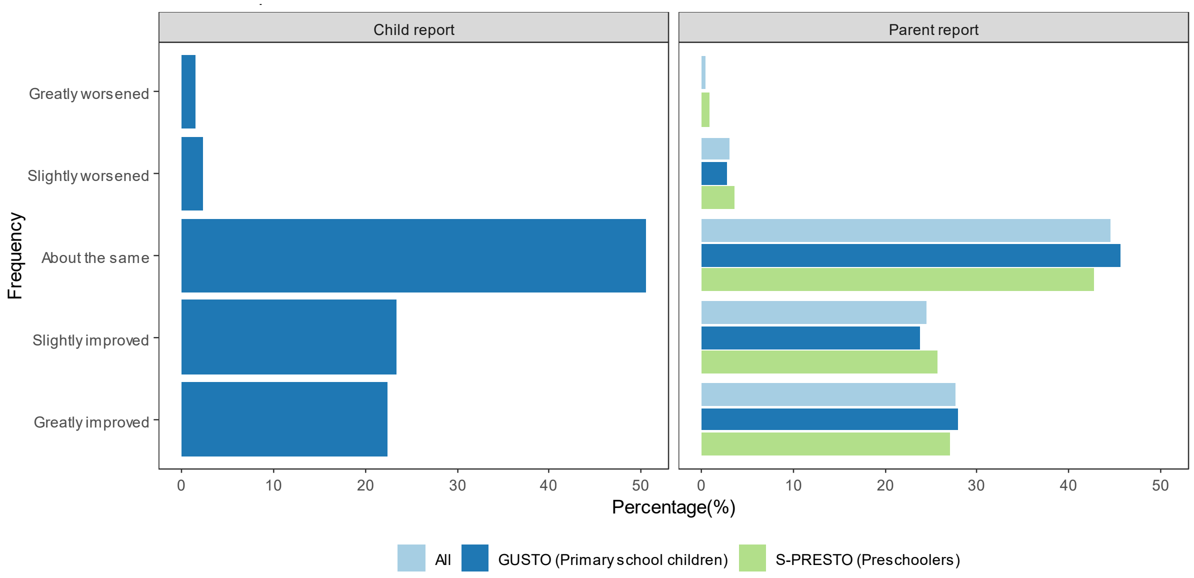

eFigure 4. Parent- and Child-Reported Child Sleep Duration in the Postlockdown Period in School-Aged Children (GUSTO) Only

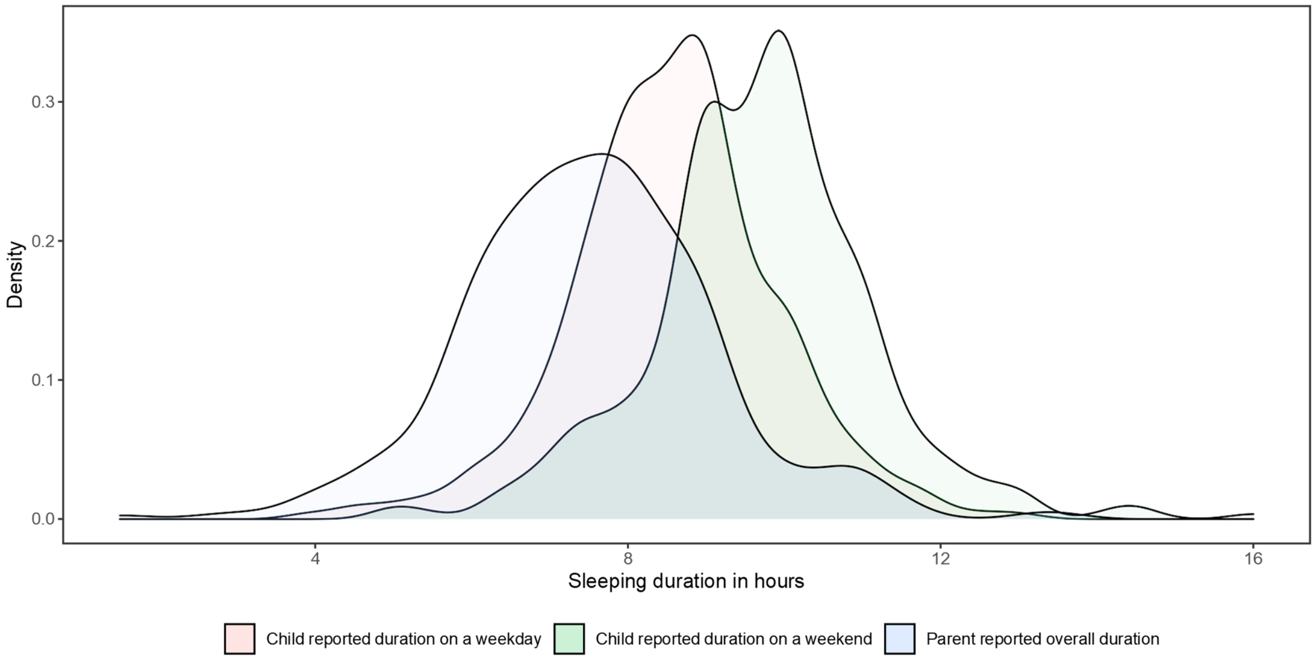

© 2022 Sum KK et al. *JAMA Pediatrics.*

eFigure 5. Parent-Reported Child Desire or Aversion to Activities Outside the Home During Lockdown

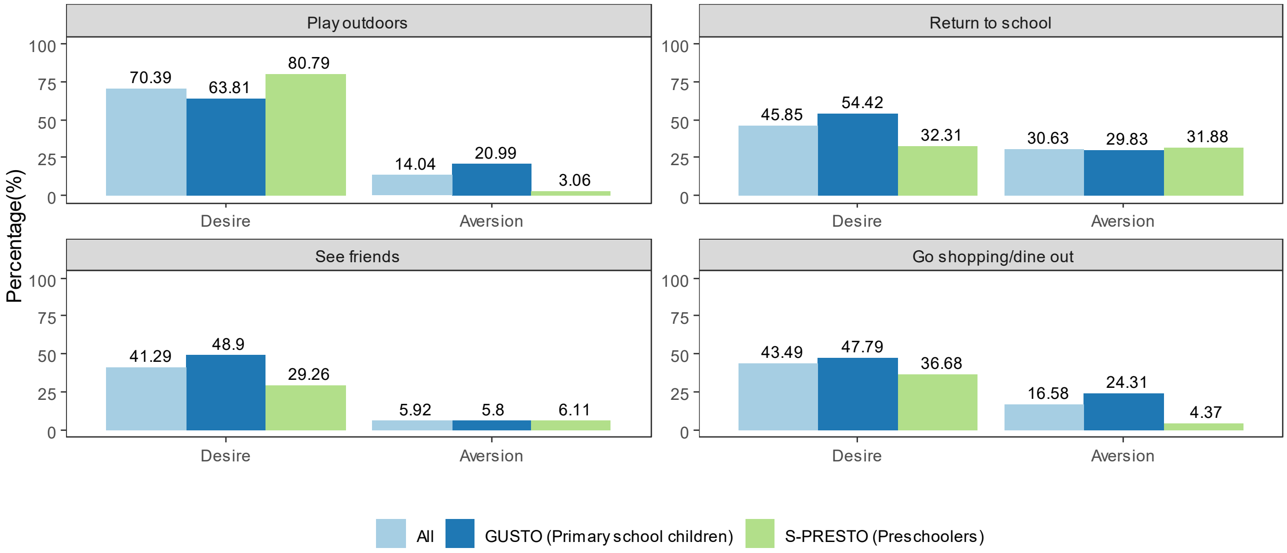

* All preferences differed by cohort (p<0.05 using Fisher's exact test) except aversion to see friends and return to school.

* Only those who answered 'yes' are displayed.

© 2022 Sum KK et al. *JAMA Pediatrics.*

eFigure 6. Average Ratings of the Parent-Reported Life Experiences in the Order of Most Negative to Most Positive

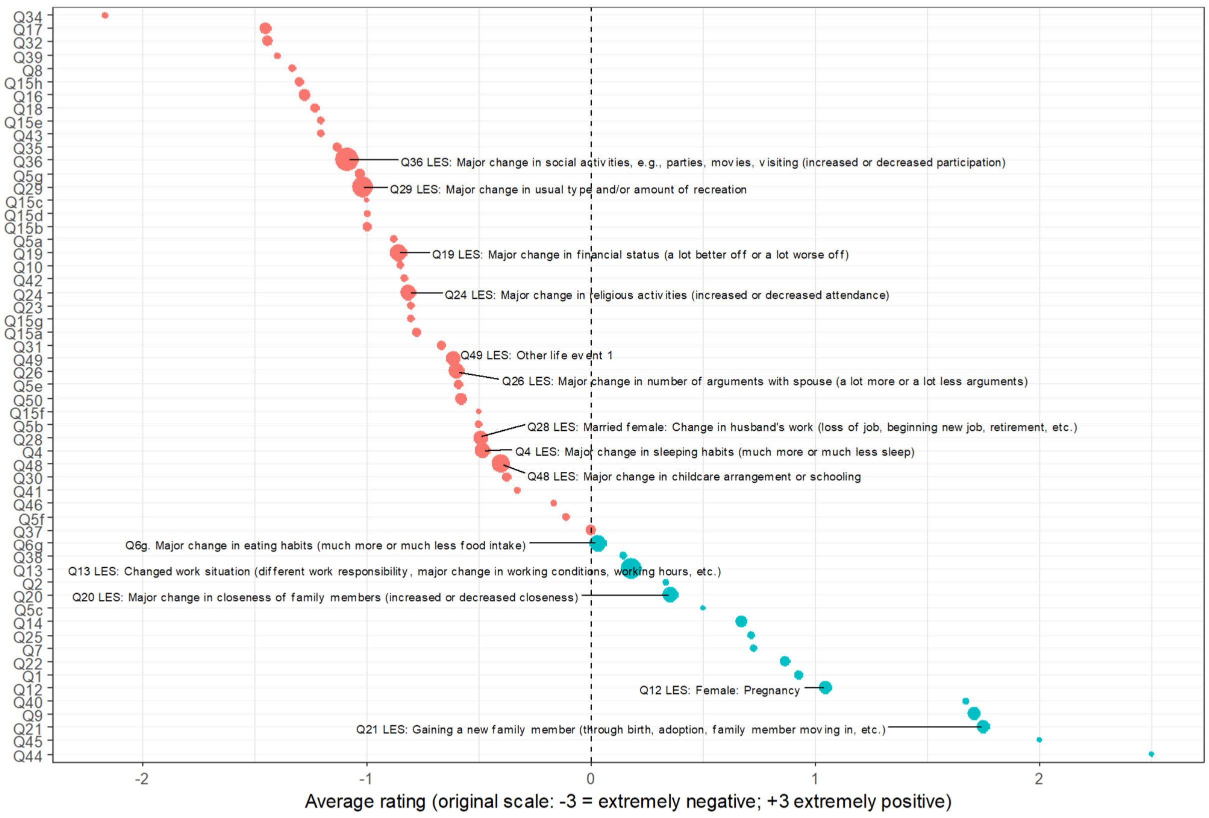

Dot sizes are proportional to number of events reported. Labelled events are those reported more than 75 times (~12.5% of participants).

© 2022 Sum KK et al. *JAMA Pediatrics.*

eFigure 7. Non-Neutral (Positive or Negative Ratings) of Major Recent Life Experiences, in Descending Order of Negative Ratings

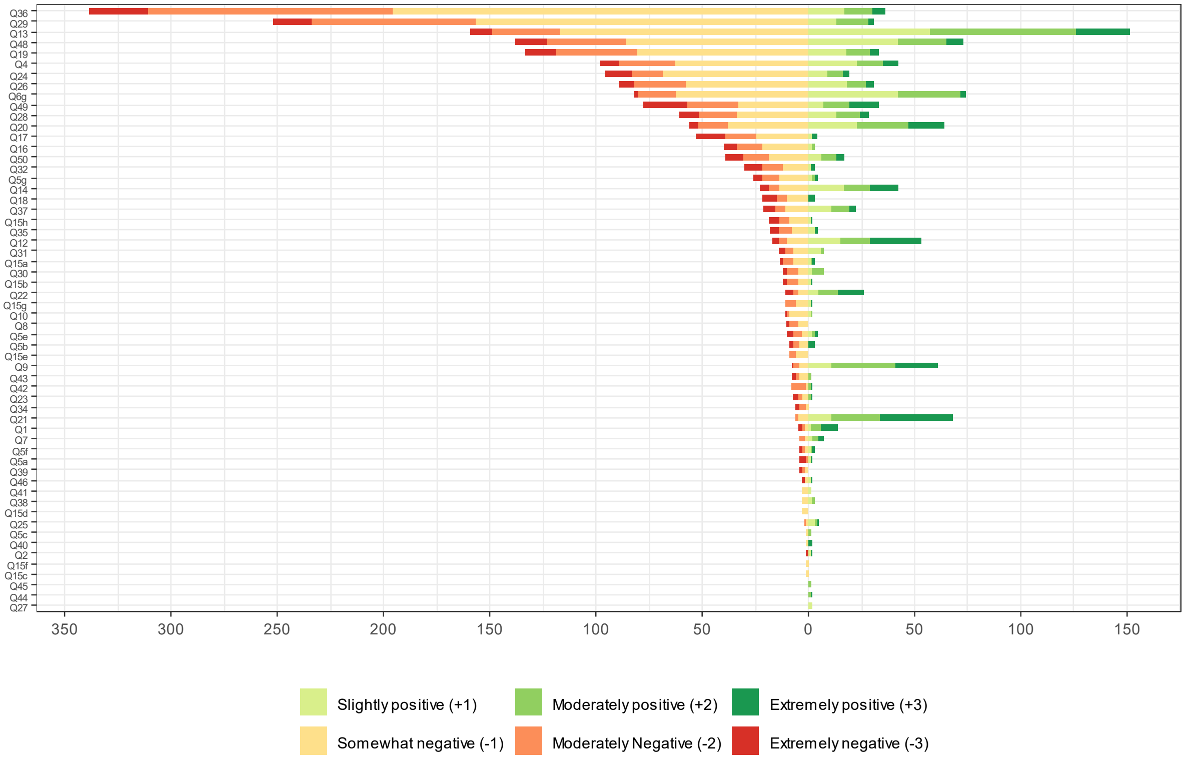

X-axis shows the number of respondents reporting the event. Many events have highly divergent ratings including Q13 ("Changed work situation"), Q6g ("Major change in eating habits"), and Q20 ("Major change in closeness of family members"). Full questions can be found in the Life Experiences Survey.

© 2022 Sum KK et al. *JAMA Pediatrics.*

eFigure 8. Association Between Prelockdown Household Income (per SGD 1000 Higher Income) and Odds of Having a Major Life Experience (yes/no), Adjusted for Cohort Membership

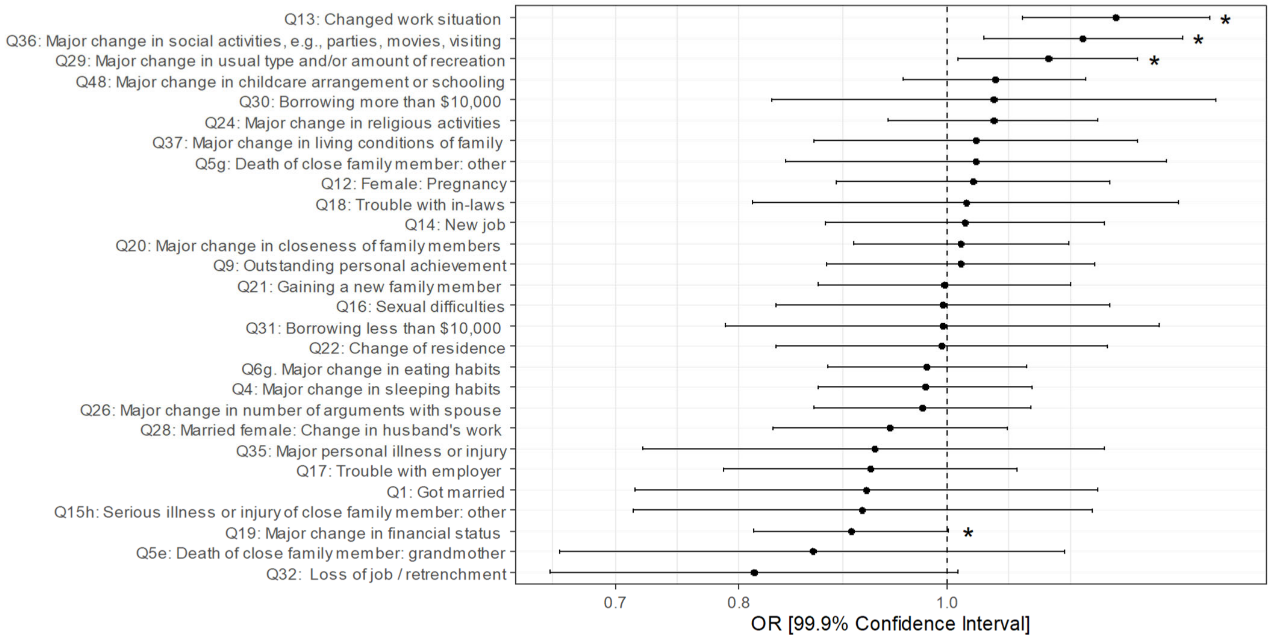

© 2022 Sum KK et al. *JAMA Pediatrics.*

eFigure 9. Association Between Prelockdown Household Income (per SGD 2000 Higher Income) and Rating of Major Life Experience Among Those Who Experienced That Event, Adjusted for Cohort Membership

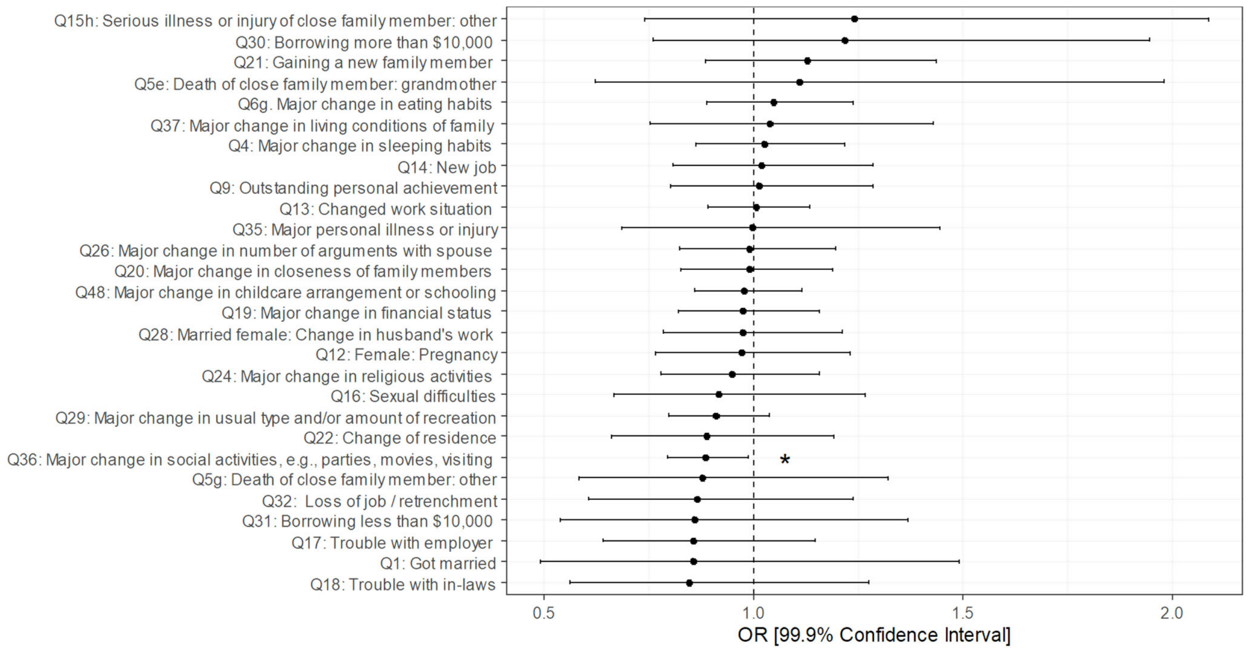

© 2022 Sum KK et al. *JAMA Pediatrics.*

eAppendix. Modified Life Experiences Survey – COVID-19

The Life Experiences Survey

Date of Interview: ☐☐ / ☐☐☐ / ☐☐☐☐
d d m m m y y y y

Person completing questionnaire:
☐ Mother ☐ Father ☐ Caregiver, relationship to GUSTO child:_______________

Listed below are a number of events which sometimes bring about change in the lives of those who experience them and which necessitate social readjustment.

- **If you *did not* experience an event *within the last year* (before the date of this survey), please mark under the 'N.A.' column.**
- **If you experienced an event, please mark whether the event occurred "0 to 6 months ago," "7 months to 1 year ago," or both.**
- **Mark "COVID-19 related?" if you believe this event was at all a result of COVID-19, include due to public safety measures such as social distancing or the "circuit breaker" or the global economic downturn.**

Also, for each item checked below, *please indicate the extent to which you viewed the event as having either a positive or negative impact on your life* **at the time the event occurred. That is,** *indicate the type and extent of impact that the event had*:

- **A rating of -3 would indicate an extremely negative impact.**
- **A rating of 0 suggests no impact either positive or negative.**
- **A rating of +3 would indicate an extremely positive impact.**

Be sure that all marks are directly across from the event they correspond to.

	N.A.	0 to 6 months ago	7 months to 1 year ago	COVID-19 related?	Extremely negative	Moderately negative	Somewhat negative	No impact	Slightly positive	Moderately positive	Extremely positive
1. Got married					-3	-2	-1	0	+1	+2	+3
2. Detention in jail					-3	-2	-1	0	+1	+2	+3
3. Death of spouse					-3	-2	-1	0	+1	+2	+3
4. Major change in sleeping habits (much more or much less sleep)					-3	-2	-1	0	+1	+2	+3
5. Death of close family member: a. mother					-3	-2	-1	0	+1	+2	+3
b. father					-3	-2	-1	0	+1	+2	+3
c. brother					-3	-2	-1	0	+1	+2	+3
d. sister					-3	-2	-1	0	+1	+2	+3
e. grandmother					-3	-2	-1	0	+1	+2	+3
f. grandfather					-3	-2	-1	0	+1	+2	+3
g. other (specify:______________)					-3	-2	-1	0	+1	+2	+3
6. Major change in eating habits (much more or much less food intake)					-3	-2	-1	0	+1	+2	+3
7. Foreclosure on mortgage or loan					-3	-2	-1	0	+1	+2	+3
8. Death of close friend					-3	-2	-1	0	+1	+2	+3

© 2022 Sum KK et al. *JAMA Pediatrics.*

9. Outstanding personal achievement					-3	-2	-1	0	+1	+2	+3
	N.A.	0 to 6 months ago	7 months to 1 year ago	COVID-19 related?	Extremely negative	Moderately negative	Somewhat negative	No impact	Slightly positive	Moderately positive	Extremely positive
10. Minor law violations (traffic tickets, disturbing the peace, etc.)					-3	-2	-1	0	+1	+2	+3
11. *Male*: Wife/girlfriend's pregnancy					-3	-2	-1	0	+1	+2	+3
12. *Female*: Pregnancy					-3	-2	-1	0	+1	+2	+3
13. Changed work situation (different work responsibility, major change in working conditions, working hours, etc.)					-3	-2	-1	0	+1	+2	+3
14. New job					-3	-2	-1	0	+1	+2	+3
15. Serious illness or injury of close family member: a. mother					-3	-2	-1	0	+1	+2	+3
b. father					-3	-2	-1	0	+1	+2	+3
c. brother					-3	-2	-1	0	+1	+2	+3
d. sister					-3	-2	-1	0	+1	+2	+3
e. grandmother					-3	-2	-1	0	+1	+2	+3
f. grandfather					-3	-2	-1	0	+1	+2	+3
g. spouse					-3	-2	-1	0	+1	+2	+3
h. other (specify:______________)					-3	-2	-1	0	+1	+2	+3
16. Sexual difficulties					-3	-2	-1	0	+1	+2	+3
17. Trouble with employer (in danger of losing job, being suspended, demoted, etc.)					-3	-2	-1	0	+1	+2	+3
18. Trouble with in-laws					-3	-2	-1	0	+1	+2	+3
19. Major change in financial status (a lot better off or a lot worse off)					-3	-2	-1	0	+1	+2	+3
20. Major change in closeness of family members (increased or decreased closeness)					-3	-2	-1	0	+1	+2	+3
21. Gaining a new family member (through birth, adoption, family member moving in, etc.)					-3	-2	-1	0	+1	+2	+3
22. Change of residence					-3	-2	-1	0	+1	+2	+3
23. Marital separation (due to conflict)					-3	-2	-1	0	+1	+2	+3
24. Major change in religious activities (increased or decreased attendance)					-3	-2	-1	0	+1	+2	+3

© 2022 Sum KK et al. *JAMA Pediatrics.*

25. Marital reconciliation					-3	-2	-1	0	+1	+2	+3

© 2022 Sum KK et al. *JAMA Pediatrics.*

	N.A.	0 to 6 months ago	7 months to 1 year ago	COVID-19 related?	Extremely negative	Moderately negative	Somewhat negative	No impact	Slightly positive	Moderately positive	Extremely positive
26. Major change in number of arguments with spouse (a lot more or a lot less arguments)					-3	-2	-1	0	+1	+2	+3
27. *Married male*: Change in wife's work outside the home (beginning work, ceasing work, changing to a new job, etc.)					-3	-2	-1	0	+1	+2	+3
28. *Married female*: Change in husband's work (loss of job, beginning new job, retirement, etc.)					-3	-2	-1	0	+1	+2	+3
29. Major change in usual type and/or amount of recreation					-3	-2	-1	0	+1	+2	+3
30. Borrowing more than $10,000 (buying home, business, etc.)					-3	-2	-1	0	+1	+2	+3
31. Borrowing less than $10,000 (buying car, TV, getting school loan, etc.)					-3	-2	-1	0	+1	+2	+3
32. Loss of job / retrenchment					-3	-2	-1	0	+1	+2	+3
33. *Male*: Wife/girlfriend having abortion					-3	-2	-1	0	+1	+2	+3
34. *Female*: Having abortion					-3	-2	-1	0	+1	+2	+3
35. Major personal illness or injury					-3	-2	-1	0	+1	+2	+3
36. Major change in social activities, e.g., parties, movies, visiting (increased or decreased participation)					-3	-2	-1	0	+1	+2	+3
37. Major change in living conditions of family (building new home, remodeling, deterioration of home, neighborhood, etc.)					-3	-2	-1	0	+1	+2	+3
38. Divorce					-3	-2	-1	0	+1	+2	+3
39. Serious injury or illness of close friend					-3	-2	-1	0	+1	+2	+3
40. Retirement from work					-3	-2	-1	0	+1	+2	+3
41. Son or daughter leaving home (due to marriage, college, etc.)					-3	-2	-1	0	+1	+2	+3
42. Ending of formal schooling					-3	-2	-1	0	+1	+2	+3

© 2022 Sum KK et al. *JAMA Pediatrics.*

	N.A.	0 to 6 months ago	7 months to 1 year ago	COVID-19 related?	Extremely negative	Moderately negative	Somewhat negative	No impact	Slightly positive	Moderately positive	Extremely positive
43. Separation from spouse (due to work, travel, etc.)					-3	-2	-1	0	+1	+2	+3
44. Engagement					-3	-2	-1	0	+1	+2	+3
45. Breaking up with boyfriend/girlfriend					-3	-2	-1	0	+1	+2	+3
46. Leaving home for the first time					-3	-2	-1	0	+1	+2	+3
47. Reconciliation with boyfriend/girlfriend					-3	-2	-1	0	+1	+2	+3
48. Major change in childcare arrangement or schooling					-3	-2	-1	0	+1	+2	+3
Other recent experiences which have had an impact on your life. List and rate.											
49. ___________________________					-3	-2	-1	0	+1	+2	+3
50. ___________________________					-3	-2	-1	0	+1	+2	+3

© 2022 Sum KK et al. *JAMA Pediatrics.*
